# Supplementary material for: De novo genome assembly and comparative genomics for the colonial ascidian Botrylloides violaceus
Source: G3 (Bethesda). 2023 Aug 9;13(10):jkad181. doi: 10.1093/g3journal/jkad181 (PMC10542563; doi:10.1093/g3journal/jkad181)
Supplement: jkad181_Supplementary_Data [file jkad181_supplementary_data.zip › Supplemental_Figure_Legends_G3-2023-404199.docx]

**Supplemental Figure Legends:**

**Supplementary Figure S1:** Determining optimal number of clusters, *k*, by identifying local maxima of the silhouette score.

**Supplementary Figure S2:** Pairwise correlation analysis of gene counts per orthogroup.

**Supplementary Figure S3:** Orthogroup overlap between tunicate species.

**Supplementary Figure S4:** KEGG enrichment of HOG clusters.
