# Supplementary figures and images for: De novo genome assembly and comparative genomics for the colonial ascidian Botrylloides violaceus
Source: G3 (Bethesda). 2023 Aug 9;13(10):jkad181. doi: 10.1093/g3journal/jkad181 (PMC10542563; doi:10.1093/g3journal/jkad181)

Supplemental Figure S1:

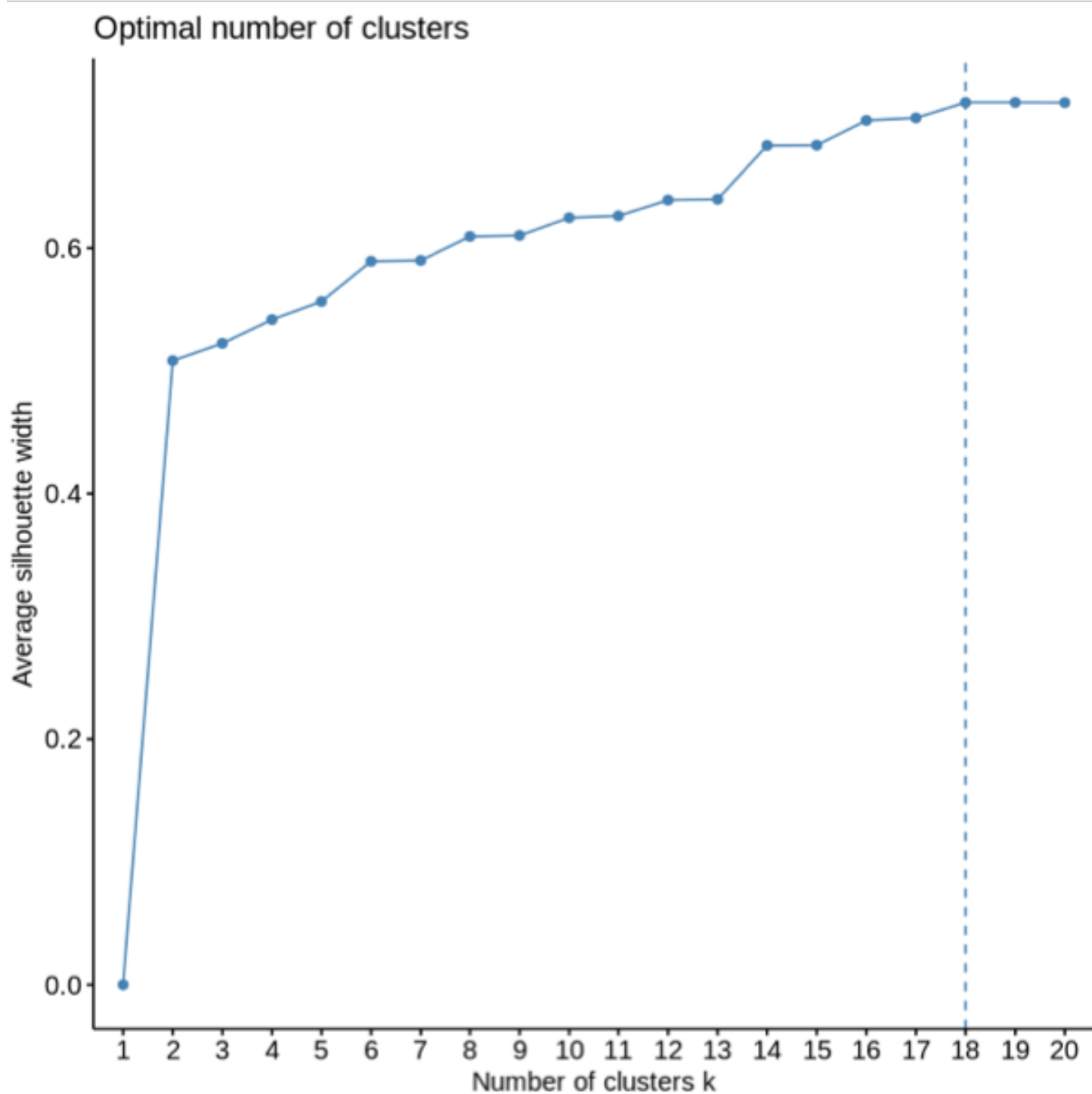

Supplemental Figure S2:

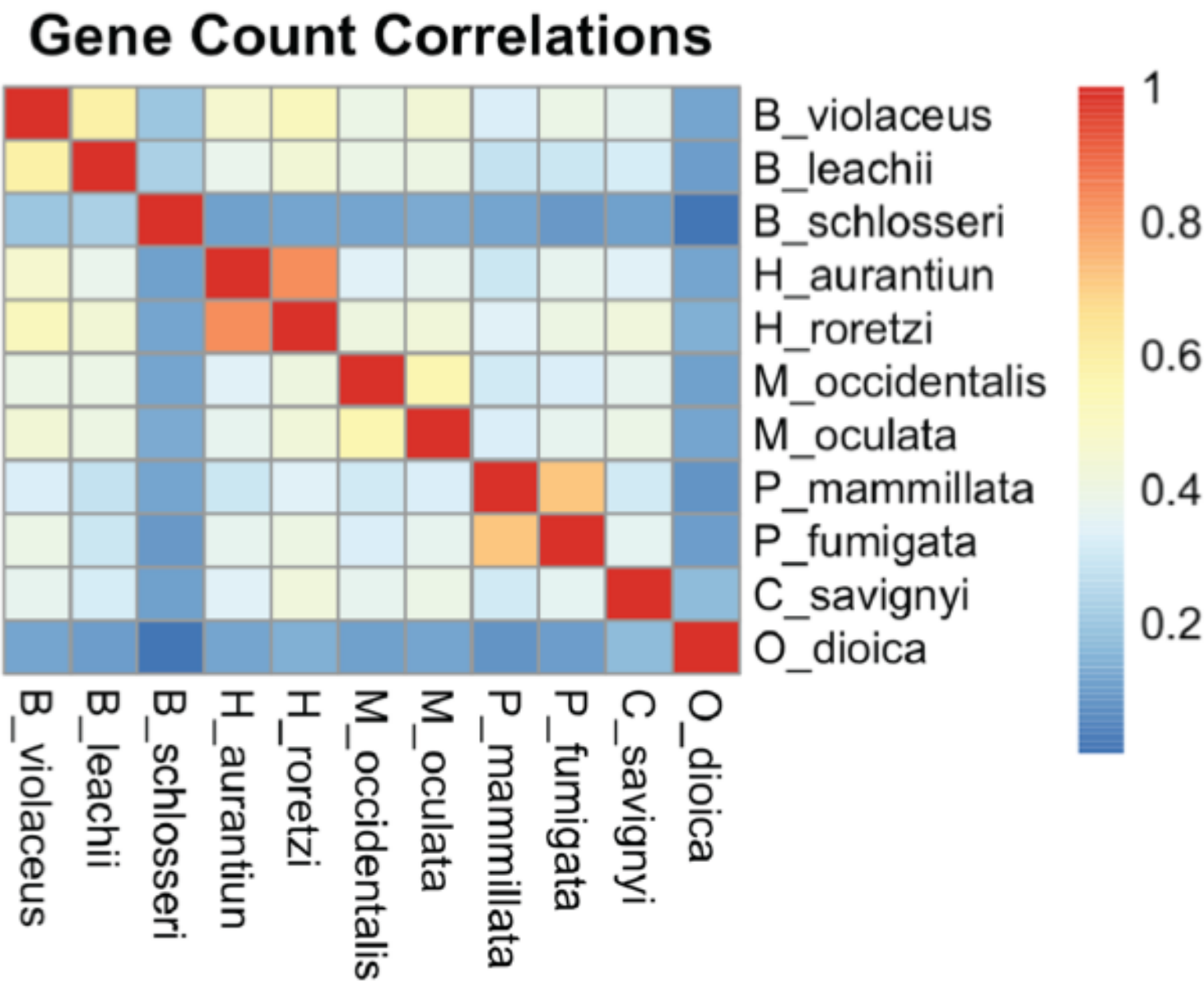

Supplementary Figure S3:

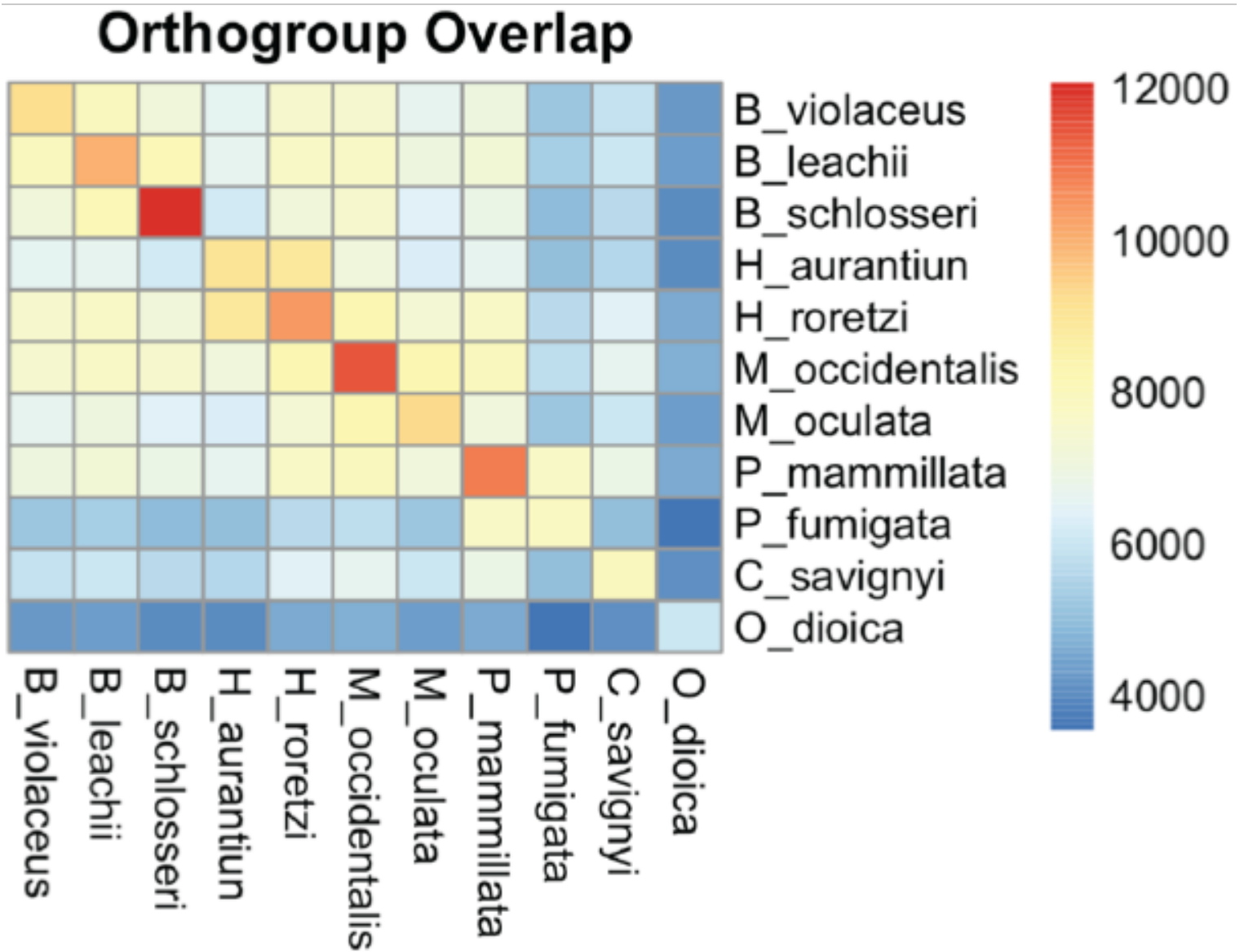

Supplementary Figure S4:

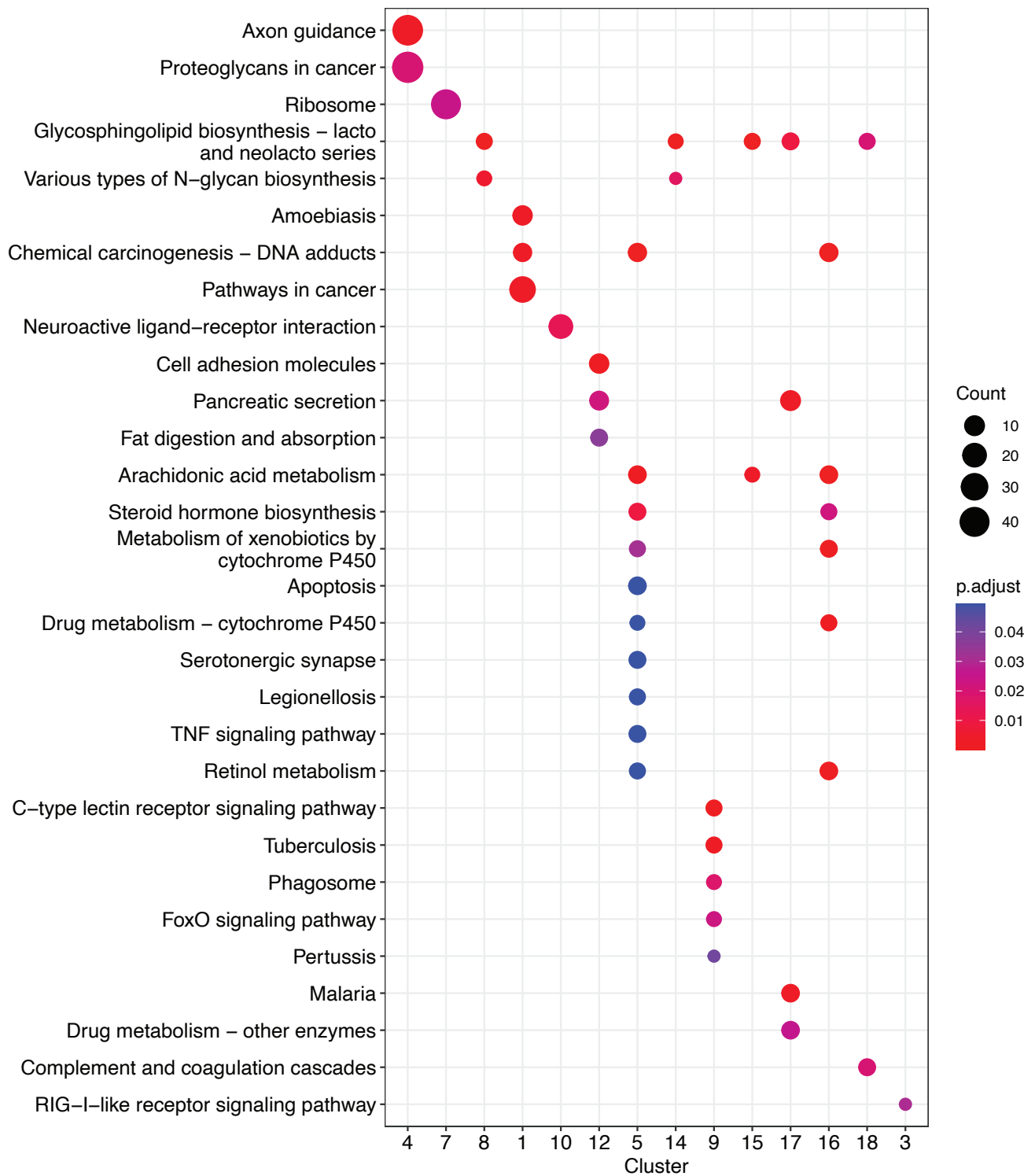

Supplement: jkad181_Supplementary_Data [file jkad181_supplementary_data.zip › Supplemental_Figures_G3-2023-404199.pdf]
